# Sphenoid fungal rhinosinusitis across the disease spectrum: a case series of non-invasive fungal ball and fatal acute invasive fungal rhinosinusitis

**DOI:** 10.1097/RC9.0000000000000749

**Published:** 2026-07-31

**Authors:** Ghada A. AlAnsari, Raniya A. AlAnsari, Ahmed Alanzi, Rizam Alghamdi, Adnan F. Almalki, Abdulmonem A. Alsaleh

**Affiliations:** a Department of Neurosurgery, King Fahad University Hospital, Imam Abdulrahman Bin Faisal University, Dammam, Saudi Arabia; b Department of Medical Laboratory Science, Mohammed Al-Mana College for Medical Sciences, Dammam, Saudi Arabia; c Anaesthesia and Pain Management Department, King Hamad University Hospital, Muharraq, Kingdom of Bahrain; d Department of ENT, Dammam Medical Complex, Dammam, Saudi Arabia; e Basic Sciences Department, College of Sciences and Health Professions, King Saud bin Abdulaziz University for Health Sciences (KSAU-HS), Ministry of National Guard - Health Affairs (MNGHA), Riyadh, Saudi Arabia; f Blood and Cancer Research Department, King Abdullah International Medical Research Center (KAIMRC), Ministry of National Guard - Health Affairs (MNGHA), Riyadh, Saudi Arabia

**Keywords:** acute invasive fungal rhinosinusitis, fungal ball, isolated sphenoid sinus disease, mucormycosis, rhino-orbital-cerebral infection, sphenoid fungal rhinosinusitis

## Abstract

**Introduction:**

Sphenoid fungal rhinosinusitis is an uncommon but clinically important entity ranging from non-invasive fungal ball to rapidly fatal acute invasive fungal rhinosinusitis.

**Case presentation:**

We report two contrasting cases of sphenoid fungal disease. The first was a 28-year-old immunocompetent woman with chronic unilateral refractory headache caused by an isolated sphenoid fungal ball, which was successfully treated with endoscopic sphenoidotomy and clearance. The second was a 49-year-old man with diabetes mellitus who presented with orbital involvement, cavernous sinus thrombosis, and fungal encephalitis due to an invasive Rhizopus infection, despite urgent debridement and systemic amphotericin B, with a fatal outcome.

**Discussion:**

These cases illustrate how sphenoid fungal disease may present either as an isolated refractory headache in an otherwise healthy patient or as a fulminant invasive infection in a high-risk host. Early imaging, endoscopic assessment, and timely differentiation between non-invasive and invasive disease are essential because management and prognosis differ markedly.

**Conclusion:**

The paired presentation highlights key red flags for early diagnosis and emphasizes the need for prompt multidisciplinary management, especially in diabetic or immunocompromised patients.

## Introduction

Isolated sphenoid sinus disease is uncommon but clinically important because of the close anatomical relationship of the sphenoid sinus to the optic nerve, cavernous sinus, internal carotid artery, skull base, and cranial nerves. Pathology in this region may therefore present with non-specific symptoms but can progress to serious orbital, vascular, or intracranial complications if diagnosis is delayed^[^1^]^.

HIGHLIGHTS

- This case report illustrates the contrasting extremes of sphenoid fungal rhinosinusitis, from an indolent fungal ball to a fatal invasive rhino-orbital-cerebral infection.
- A persistent unilateral refractory headache may be the only presenting feature of isolated sphenoid fungal disease.
- Early computed tomography or magnetic resonance imaging is crucial when headache is atypical, severe, or unresponsive to standard therapy.
- Diabetes, orbital findings, necrotic nasal tissue, and neurologic deterioration are major red flags for acute invasive fungal rhinosinusitis.
- Prompt recognition is essential because a localized fungal ball is usually curable surgically, whereas invasive disease may be rapidly fatal despite aggressive treatment.

Fungal rhinosinusitis involving the sphenoid sinus represents a rare but important subgroup and may occur across a wide clinical spectrum. At one end, a non-invasive fungal ball typically affects immunocompetent patients, follows an indolent course, and is usually curable with endoscopic surgical clearance^[^2^]^. At the other end, acute invasive fungal rhinosinusitis is more frequently encountered in patients with diabetes mellitus or immunocompromised states and may rapidly progress to rhino-orbital-cerebral infection, cavernous sinus thrombosis, fungal encephalitis, and death^[^3^]^.

Diagnosis can be challenging because early symptoms are often vague. Persistent unilateral or retro-orbital headache may be the only presenting symptom of an isolated sphenoid fungal ball, often without nasal obstruction, rhinorrhea, or abnormal nasal endoscopy^[^4^]^. In contrast, visual loss, ophthalmoplegia, proptosis, necrotic nasal tissue, cranial neuropathies, or altered consciousness should raise urgent concern for invasive fungal disease, especially in high-risk patients^[^5^]^.

Computed tomography is useful for detecting sphenoid opacification, intralesional hyperdensity or calcification, and bony remodeling, whereas magnetic resonance imaging is essential when orbital, vascular, or intracranial extension is suspected^[^3,6^]^. We present two contrasting cases of sphenoid fungal rhinosinusitis to highlight the diagnostic and prognostic differences between a localized non-invasive fungal ball and fulminant invasive disease. This case report was prepared in line with the SCARE guidelines^[^7^]^.

## Case presentation

### Case 1

A 28-year-old immunocompetent woman with no known chronic medical illness presented to the otolaryngology clinic with a 4-month history of persistent, severe left-sided periocular headache. The pain was described as debilitating and electric shock-like in quality. She denied nasal obstruction, rhinorrhea, epistaxis, visual disturbance, or focal neurological symptoms.

Before referral, she had been evaluated at another institution and treated for presumed migraine-related status migrainosus. She was admitted for analgesia and discharged on migraine-directed therapy; however, her symptoms persisted without meaningful improvement. Oral analgesics provided only partial relief, and during the 2 weeks before presentation she required intravenous analgesia for adequate pain control. The strictly unilateral, prolonged, and refractory nature of the headache made a secondary structural cause more likely. Cluster headache was considered less likely because there was no documented attack periodicity or cranial autonomic symptoms, and trigeminal neuralgia was considered unlikely because the pain was persistent rather than brief, triggerable paroxysms.

**Figure 1.**
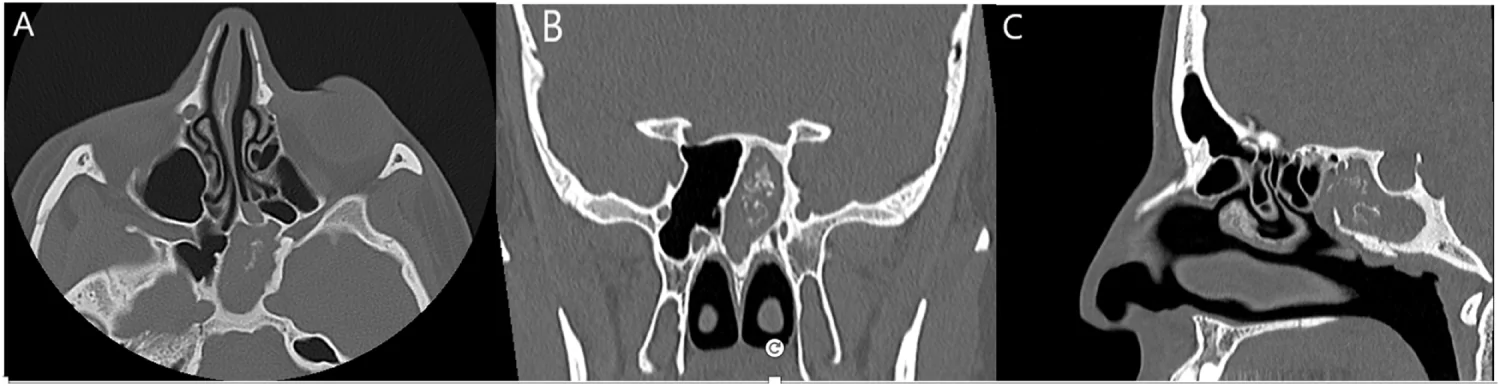
Non-enhanced CT scans of paranasal sinuses. (A) Axial view showing complete homogeneous opacification of the left sphenoid sinus. The contents show heterogeneous hyperdensity. (B) Coronal view showing wall thickening and sclerosis with central calcification of the left sphenoid sinus cavity. (C) Sagittal view showing left sphenoid sinus expansion with homogeneous opacification extending to the sphenoethmoidal recess and wall hyperostosis. There is no erosion into the sella, clivus, cavernous sinus region, or carotid canal.

Nasal endoscopy was unremarkable, and routine hematologic investigations were within normal limits. Non-enhanced computed tomography of the paranasal sinuses demonstrated isolated left sphenoid sinus disease. Axial images showed complete opacification of the left sphenoid sinus with heterogeneous hyperdense contents. Coronal images demonstrated wall thickening, sclerosis, and central calcification within the sinus cavity. Sagittal images showed expansion of the left sphenoid sinus with opacification extending to the sphenoethmoidal recess and associated wall hyperostosis. There was no radiologic evidence of erosion into the sella, clivus, cavernous sinus region, or carotid canal. These findings favored a non-invasive isolated sphenoid fungal ball rather than invasive fungal disease or an intracranial process (Fig. 1).

The patient underwent transnasal endoscopic sphenoidotomy. Dense fungal debris filling the left sphenoid sinus was identified and completely removed, with no reported intraoperative complications. Histopathological examination showed fibro-collagenous tissue with a chronic inflammatory infiltrate admixed with fungal elements, consistent with fungal mycetoma, without evidence of tissue invasion. Organism-level fungal characterization was not available in the accessible record. The postoperative course was uneventful, and the patient experienced complete resolution of headache. At 6-month outpatient follow-up, she remained symptom-free.

### Case 2

A 49-year-old man with diabetes mellitus and hypertension presented to the emergency department with a 1-week history of sudden-onset, severe, intermittent headache associated with progressive visual loss in the left eye. His symptoms were accompanied by generalized fatigue, dizziness, left-sided proptosis, and partial ptosis, which progressed to complete ptosis over the following days. He subsequently developed generalized weakness and a progressive deterioration in consciousness.

On arrival, his Glasgow Coma Scale score was 8/15. Examination of the left eye revealed proptosis, complete ptosis, conjunctival chemosis, and a fixed, dilated pupil. Necrotic-appearing tissue extending from the left nasal cavity was also noted. In the setting of diabetes mellitus, rapidly progressive orbital signs, visual loss, necrotic nasal tissue, and reduced consciousness, acute invasive fungal rhinosinusitis with orbital and intracranial extension was suspected.

**Figure 2.**
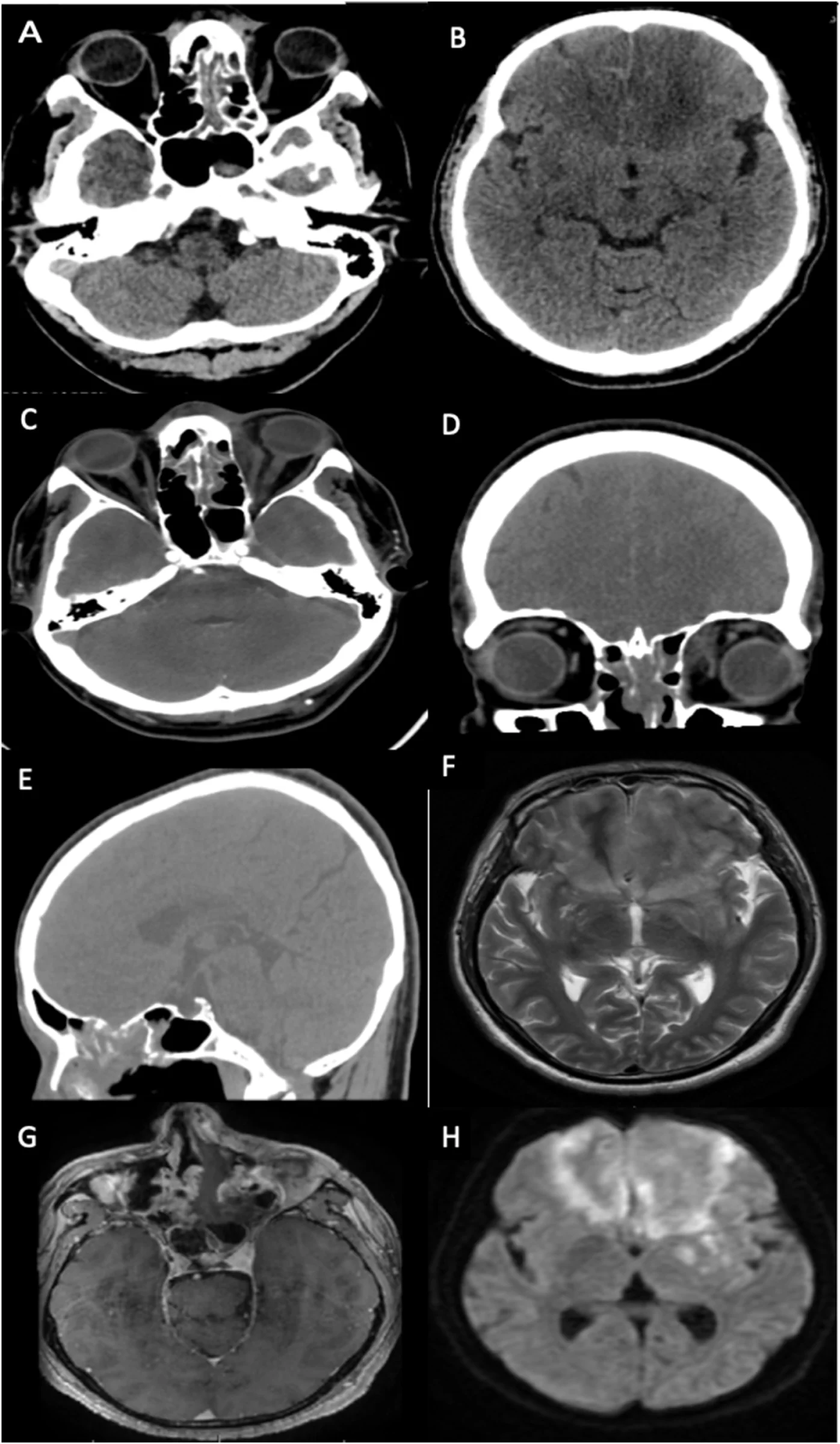
Non-enhanced brain CT scans, soft-tissue axial views. (A) Opacification of the left frontal sinus, sphenoid sinus, and ethmoid air cells. (B) Bifrontal ill-defined hypodensities extending anteriorly and laterally, producing a mild mass effect with an early left-to-right midline shift. Bone window images: (C) axial view, (D) coronal view, and (E) sagittal view, showing homogeneous opacification of the left frontal sinus, sphenoid sinus, and ethmoid air cells with hyperdense calcifications. There is an erosion of the lamina papyracea (medial orbital wall) on the left side and an extension of fungal elements into the medial extraconal orbital space. The left optic canal exhibits signs of thinning and partial dehiscence, indicating involvement of the optic nerve. The left orbital fat appears less well-defined and strandy, indicating orbital cellulitis. Magnetic resonance imaging (MRI) of the brain with contrast, axial views. (F) T2W image showing hyperintense edematous changes in the bilateral frontal lobes and left basal ganglia with mass effect on the frontal horns of the lateral ventricles. (G) T1W post-contrast image showing left medial orbital intraconal intermediate signal intensity with no definite contrast enhancement. There is evidence of extension into the left cavernous sinus and adjacent brain parenchyma, involving the left medial temporal lobe and basal frontal lobe. Mucosal thickening of the left concha, left ethmoid, and left frontal sinuses is also noted. (H) DWI image showing diffusion restriction consistent with invasive fungal encephalitis with venous infarction due to cavernous sinus thrombosis.

Urgent computed tomography of the brain was performed (Fig. 2A–E), and nasal tissue was collected for microbiological culture and histopathological assessment. Because of rapid neurological deterioration, the patient was emergently stabilized, intubated, and transferred to the intensive care unit. Intravenous amphotericin B was initiated promptly after clinical and radiologic suspicion of invasive fungal rhinosinusitis. Urgent multidisciplinary surgical debridement was performed by otolaryngology, neurosurgery, and ophthalmology teams, with the aim of reducing fungal burden and limiting further disease extension.

Subsequent magnetic resonance imaging of the brain further demonstrated the extent of rhino-orbital-cerebral invasion (Fig. 2F–H). Microbiological culture ultimately confirmed *a Rhizopus* species infection. The final clinicoradiologic diagnosis was acute invasive fungal rhinosinusitis complicated by left orbital involvement, cavernous sinus thrombosis, and fungal encephalitis, with cavernous sinus involvement later supported by magnetic resonance venography. Despite aggressive multidisciplinary management, including systemic amphotericin B therapy, intensive care support, and urgent surgical debridement, the disease progressed rapidly and the patient died.

**Figure 3.**
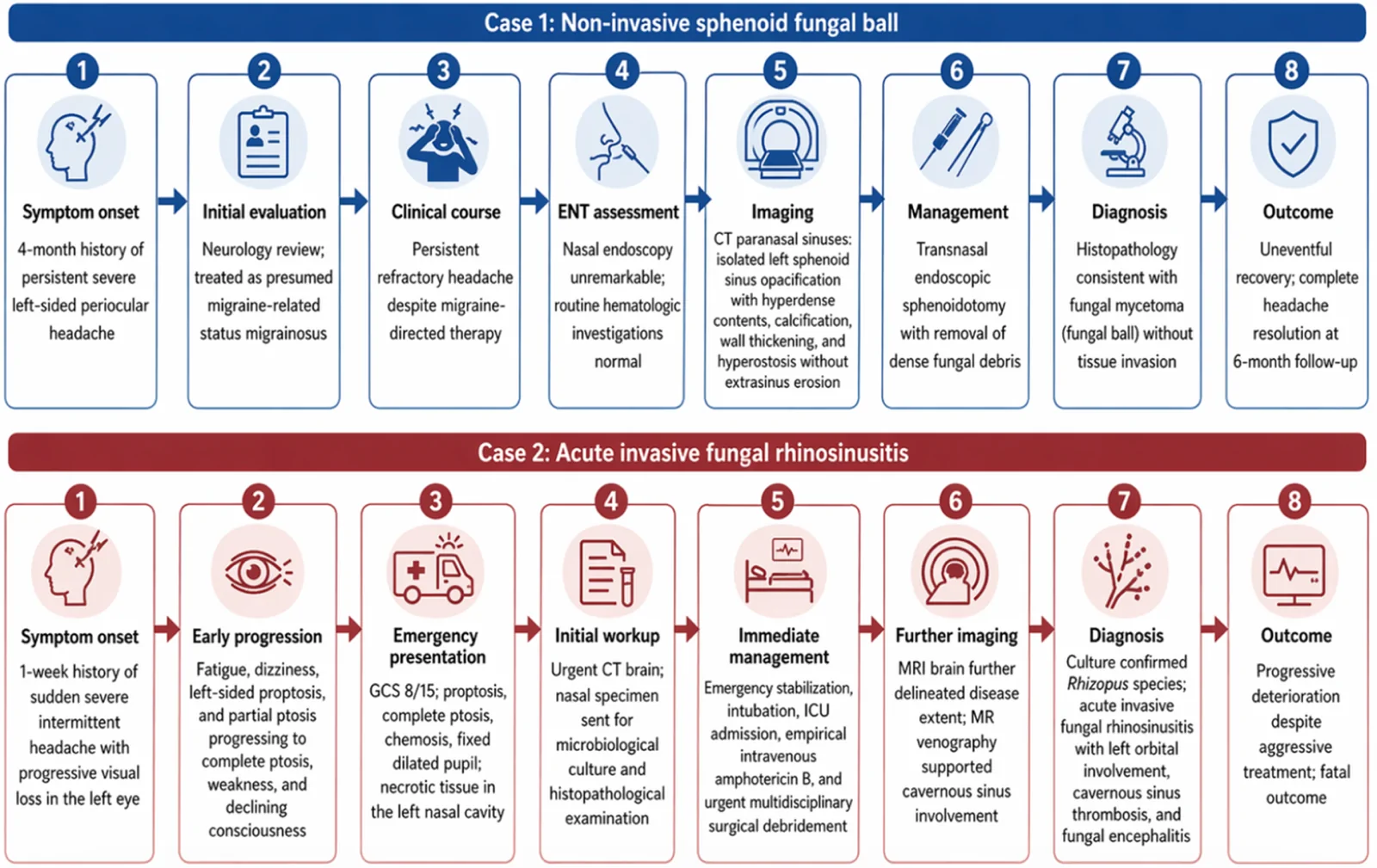
Timeline summarizing the clinical course, diagnostic workup, management, and outcomes of the two cases of sphenoid fungal rhinosinusitis.

The chronological progression, diagnostic workup, management, and outcomes of both cases are summarized in Figure 3.

## Discussion

### Clinical spectrum and educational value

Sphenoid fungal rhinosinusitis is uncommon but clinically important because it may range from an indolent, non-invasive fungal ball to fulminant acute invasive fungal rhinosinusitis. The educational value of this report lies in presenting two contrasting outcomes from the same anatomical site. In the first case, the disease occurred in an immunocompetent patient, remained localized, and was cured surgically. In the second case, diabetes mellitus was associated with rapidly progressive rhino-orbital-cerebral mucormycosis, cavernous sinus thrombosis, fungal encephalitis, and death despite urgent multidisciplinary treatment. Similar reports of isolated sphenoidal fungal disease and invasive sphenoid fungal sinusitis emphasize that clinical behavior depends strongly on host risk factors, symptom tempo, radiologic pattern, and evidence of tissue invasion^[^8–10^]^.

### Case 1 in context: diagnostic pitfall of non-invasive disease

The first case reflects a well-recognized diagnostic pitfall of isolated sphenoid fungal ball. Patients may present primarily with persistent unilateral or retro-orbital headache, while nasal obstruction, rhinorrhea, and abnormal nasal endoscopy may be absent^[^4^]^. This explains why such patients are often initially assessed in neurology settings before sinonasal imaging is obtained^[^4^]^. In our patient, the prolonged refractory periocular headache, absence of sinonasal symptoms, normal nasal endoscopy, and computed tomography (CT) findings of isolated sphenoid opacification with hyperdense material, calcification, wall thickening, and hyperostosis were consistent with a non-invasive fungal ball. Similar clinical and radiologic patterns have been described in isolated sphenoid fungal ball, isolated sphenoid inflammatory disease, and isolated sphenoid sinusitis presenting mainly as headache^[^11–13^]^. Tertiary-center experience with sphenoid fungal ball also supports the role of CT in identifying localized fungal disease and guiding endoscopic surgical management^[^14^]^.

### Case 2 in context: fulminant invasive progression

The second case illustrates the fulminant end of the disease spectrum. Acute invasive fungal rhinosinusitis is strongly associated with diabetes mellitus and other immunocompromised states, and may progress through angioinvasion, tissue necrosis, vascular thrombosis, orbital invasion, and intracranial spread^[^3^]^. Our patient had multiple red flags, including diabetes mellitus, severe headache, visual loss, proptosis, ptosis, chemosis, fixed, dilated pupil, necrotic nasal tissue, and reduced consciousness. Invasive sphenoid fungal disease may mimic sellar or skull-base pathology when neurological or ophthalmic features dominate, which can delay recognition^[^15^]^. Imaging reviews of rhino-orbito-cerebral fungal sinusitis further emphasize the importance of detecting extrasinus extension, orbital involvement, vascular complications, and intracranial spread^[^16^]^. Reports of invasive fungal sphenoid sinusitis complicated by bone erosion and meningoencephalitis similarly highlight the poor prognosis once central nervous system involvement occurs^[^17^]^.

### Management and prognostic implications

Management differs fundamentally between non-invasive fungal ball and acute invasive fungal rhinosinusitis. For non-invasive sphenoid fungal ball, endoscopic sphenoidotomy with complete removal of fungal debris is generally effective, and recurrence is uncommon when adequate surgical clearance and ventilation are achieved^[^2^]^. This was reflected in our first patient, whose headache resolved completely after endoscopic clearance. In contrast, acute invasive fungal rhinosinusitis requires immediate combined treatment with systemic antifungal therapy, aggressive surgical debridement, intensive supportive care, and correction of predisposing metabolic abnormalities when possible^[^3,10^]^. Recognition of microinvasive or early invasive patterns may be important because fungal rhinosinusitis can exist along a continuum rather than as a simple non-invasive versus invasive binary distinction^[^18^]^. Selected severe rhino-orbital-cerebral cases may also require adjunctive orbital-directed therapy in addition to endoscopic debridement and systemic antifungal treatment^[^19^]^. Larger surgical series of chronic sphenoid sinusitis support transnasal sphenoidotomy as an effective approach for appropriately selected sphenoid sinus disease^[^20^]^.

### Clinical message and practical implications

Together, these cases provide a practical diagnostic contrast. In immunocompetent patients, chronic unilateral refractory headache with isolated sphenoid opacification should prompt consideration of a fungal ball, even when nasal examination is normal. In diabetic or immunocompromised patients, headache accompanied by visual loss, ophthalmoplegia, proptosis, necrotic nasal tissue, or altered consciousness should be treated as a rhinocerebral emergency. Ocular cranial nerve palsies secondary to sphenoid sinus disease further illustrate how sphenoid pathology may initially present with neuro-ophthalmic rather than nasal symptoms^[^21^]^. Early CT can identify isolated sphenoid disease, but MRI is essential if orbital, cavernous sinus, vascular, or intracranial extension is suspected^[^3,6^]^. Prompt differentiation between non-invasive and invasive disease is critical because the former is usually surgically curable, whereas the latter may be fatal despite appropriate treatment.

### Limitations

This report has limitations. It describes only two patients from a single center, which limits generalizability. Some retrospective details were unavailable, including complete operative details, organism-level characterization in the non-invasive case, full antifungal treatment parameters, and detailed metabolic data in the invasive case. Nevertheless, the paired presentation remains clinically valuable because it demonstrates two distinct clinical trajectories of sphenoid fungal rhinosinusitis and highlights practical red flags that may help clinicians distinguish localized disease from an invasive fungal emergency.

## Conclusion

Sphenoid fungal rhinosinusitis may present as either an indolent, localized fungal ball or a rapidly fatal invasive rhino-orbital-cerebral infection. Persistent refractory unilateral or periocular headache should prompt consideration of isolated sphenoid fungal disease, even when sinonasal symptoms or endoscopic findings are absent. Conversely, visual loss, ophthalmoplegia, proptosis, necrotic nasal tissue, or neurological deterioration in a diabetic or immunocompromised patient should raise immediate suspicion of acute invasive fungal rhinosinusitis. Early recognition and prompt distinction between non-invasive and invasive disease are essential because treatment strategy and prognosis differ substantially.

## Learning points

- Sphenoid fungal rhinosinusitis ranges from an indolent non-invasive fungal ball to fulminant acute invasive fungal rhinosinusitis with potentially fatal rhino-orbital-cerebral complications.
- Persistent unilateral or periocular headache without prominent sinonasal symptoms should raise suspicion for isolated sphenoid sinus disease, especially when refractory to conventional neurological or analgesic treatment.
- CT findings suggestive of a sphenoid fungal ball include isolated sphenoid opacification, intralesional hyperdensity or calcification, wall thickening, and hyperostosis, without aggressive extrasinus erosion.
- In diabetic or immunocompromised patients, headache accompanied by visual loss, ophthalmoplegia, proptosis, necrotic nasal tissue, or altered consciousness should prompt urgent evaluation for an invasive fungal infection, as delayed recognition may be fatal despite aggressive treatment.

## Data Availability

The data supporting the findings of this case report are available from the corresponding author on reasonable request.
